# Successful Inclusion of High Vegetable Protein Sources in Feed for Rainbow Trout without Decrement in Intestinal Health

**DOI:** 10.3390/ani11123577

**Published:** 2021-12-16

**Authors:** Glenda Vélez-Calabria, David Sánchez Peñaranda, Miguel Jover-Cerdá, Silvia Martínez Llorens, Ana Tomás-Vidal

**Affiliations:** Research Group of Aquaculture and Biodiversity, Institute of Animal Science and Technology, Universitat Politècnica de València, Camino de Vera 14, 46071 València, Spain; gleveca@alumni.upv.es (G.V.-C.); dasncpea@upvnet.upv.es (D.S.P.); mjover@dca.upv.es (M.J.-C.); silmarll@dca.upv.es (S.M.L.)

**Keywords:** intestinal track, fishmeal substitution, amino acid supplement, interleukins, immune system, inflammation response

## Abstract

**Simple Summary:**

A reduction in fishmeal in diets is essential to achieve the aim of sustainable production. In the current work, using a plant protein blend of wheat gluten, wheat and soybean meal supplemented with Tau, Val, Lys and Met, a 10% higher fishmeal substitution without affecting growth and health parameters has been accomplished.

**Abstract:**

The aquaculture of carnivorous fish is in continuous expansion, which leads to the need to reduce the dependence on fishmeal (FM). Plant proteins (PP) represent a suitable protein alternative to FM and are increasingly used in fish feed. However, PP may lead to stunted growth and enteritis. In the current study, the effect of high FM substitution by PP sources on the growth, mortality and intestinal health of rainbow trout (*Oncorhynchus mykiss*) was evaluated in terms of the histological intestine parameters and expression of genes related to inflammation (*IL-1β, IL-8* and *TGF-β*) and immune responses (*Transferrin, IgT* and *IFN-γ*). The results show that a total substitution registered lower growth and survival rates, probably due to a disruption to the animal’s health. Confirming this hypothesis, fish fed FM0 showed histological changes in the intestine and gene changes related to inflammatory responses, which in the long-term could have triggered an immunosuppression. The FM10 diet presented not only a similar expression to FM20 (control diet), but also similar growth and survival. Therefore, 90% of FM substitution was demonstrated as being feasible in this species using a PP blend of wheat gluten (WG) and soybean meal (SBM) as a protein source.

## 1. Introduction

In order to improve sustainability and profitability, the aquaculture industry has followed the tendency to replace fishmeal (FM), or at least reduce it, without a decrement of nutritional quality and intestinal health [1]. As a consequence, much of the research done on carnivorous species, including rainbow trout, have been tested to evaluate the effect of FM substitution by alternative protein sources, such as soybean meal (SBM) or soy protein concentrates [2,3], rice protein concentrate or canola protein concentrate [4,5]. Alternative proteins of animal origin have also been tested, such as feather meal and meat and bone meal [6], krill [7] or bacterial protein [8], but the best growth results have been obtained with mixtures of vegetable protein ingredients [2,9,10].

Soybean protein products are considered one of the most workable alternatives due to their reasonable price and a steady supply of soybeans [11], although previous studies on high contents of soybean in diets reported alterations in the immune system of the intestinal track [12,13]. Until now, the use of SBM has been limited in feeds for salmonids, likely due to its relatively low protein content; however, concentrates produced from soybean (soybean protein concentrates; SPCs) have solved this problem with a mean crude protein (CP) content of 65–70% [14]. In fact, by including SPC as an alternative protein source it has been possible to achieve the maximum FM replacement (33 to 100% replacement) without affecting growth performance or nutrient utilization in rainbow trout [15].

Although the highest replacement has been achieved using plant proteins (PP), this protein resource presents some limitations in carnivorous species, such as the high carbohydrate content, deficiency in certain essential amino acids (EAA; e.g., methionine (Met), lysine (Lys), tryptophan (Trp), threonine (Thr) and arginine (Arg)) or low palatability. Another possible negative factor is the presence of antinutritional factors (ANFs), such as protease inhibitors, lectins, phytic acid, saponins, phytoestrogens, antivitamins and allergens [16,17]. Therefore, the inclusion of high levels of plant ingredients may affect the health conditions of fish. For example, a decrease in innate immune response and an inflammatory response upon feeding high amounts of PP ingredients has been reported in rainbow trout [18] and in other carnivorous species such as gilthead seabream, *Sparus aurata* [19,20], Senegalese sole (*Solea senegalensis*) [21] or hybrid grouper (*Epinephelus fuscoguttatus* ♀ × *Epinephelus lanceolatus* ♂) [22].

Previous studies with a total FM substitution with raw materials of vegetable origin have also been performed in carnivorous species, obtaining, during short feeding periods (64 days), similar growth rates to FM feeds [23]. However, in the long-term (>12 weeks), the growth rate decreased accompanied by high mortality associated with intestinal and hepatic histomorphological alterations [24], disorders of the immune state [20] or microbial imbalances [25]. One of main factors of this negative effect on health could be the presence of ANFs in PP. ANFs can affect the digestibility and absorption of nutrients [26], as well as intestinal integrity [27], changing the microbial abundance and species richness [25]. In contrast, the negative effects of FM substitution on health status can be partially corrected by the inclusion of EAA supplements in the diets. AA supplementation is required in feed with high FM substitution in order to restore the appropriate AA profile to the target species [28]. For example, Arg supplementation showed a significant effect on non-specific immune responses in Nile tilapia *Oreochromis niloticus* juveniles [29] and golden pompano *Trachinotus ovatus* juveniles [30]. Cheng et al. [31] evaluated the effects of a diet with Arg and glutamine (Gln) supplementation in diets for juvenile red drum (*Sciaenops ocellatus*), confirming the enhancer action of AA supplementation on the immune system as well as on intestine structure with an increase in microvillus height. In European seabass juveniles (*Dicentrarchus labrax*) [32], AA supplementation enhanced the antioxidant defense response. Finally, Feng et al. [33] reported that methionine hydroxy analogue (MHA) supplementation promoted the antioxidant defense in the intestine and hepatopancreas of juveniles Jian carp (*Cyprinus carpio* var. Jian).

In rainbow trout, high levels of PP sources or alternative protein blends (animal and vegetable) have not compromised the growth performance, even with FM substitutions higher than 75% [9,34,35,36,37,38]. Nevertheless, high FM substitutions may negatively affect fish intestinal health, as has been reported by Santigosa et al. [9] with an increase in relative intestinal length (RIL) in diets with FM substitution above 75%. Previous studies replacing FM with SBM in rainbow trout showed changes and damages to the digestive tract [3,39,40]. Romarheim et al. [41] found that rainbow trout fed a diet with 30% SBM showed a development of enteritis with a general progression of reduced mucosal fold height and increased lamina propria. Jalili et al. [42] assessed higher inclusions of other vegetable protein sources (70 and 100%; wheat gluten (WG), corn gluten (CC) and SBM), finding lower growth and depression of immune response. In other species, such as Atlantic salmon (*Salmo salar*), substitution of FM with SBM provided similar results [43]. 

In conclusion, the effect of high levels of plant ingredients on fish health conditions still remains in the process of study, and an integrative approach is needed to clarify the interactions between nutrition and the immune system in order to understand the physiological processes involved. Thus, the objective of the current study was to achieve the maximum FM substitution using as an alternative protein the mixture of PP (wheat meal (WM), WG and SBM) in rainbow trout (*Oncorhynchus mykiss*) without affecting growth, survival and intestinal health. With this aim, the intestinal health status of rainbow trout will be analyzed in terms of intestinal histology and the expression of genes related to inflammatory processes, immune systems and epithelial integrity in the anterior and posterior intestine.

## 2. Materials and Methods

The experimental protocol was reviewed and approved by the Ethics and Animal Welfare Committee of the Polytechnic University of Valencia (Official bulletin No. 80 of 06/2014), following Royal Decree 53/2013 and the European Directive 2010/63/EU on the protection of animals used for scientific research, with the purpose of minimizing the suffering of animals.

### 2.1. Production System

The growth trial was performed in a recirculation freshwater system (65 m^3^ capacity), with a rotary mechanical filter and a gravity biofilter (approximately 6 m^3^), equipped with nine cylindrical fiberglass tanks of 1750 L. All tanks were equipped with aeration. Water temperature was 15.5 ± 0.1 °C, dissolved oxygen was 9.3 ± 0.3 mg L^−1^ and pH ranged from 8.4 to 8.6. All these parameters were measured on a daily basis from Monday to Saturday. Photoperiod was natural, and all tanks had similar light conditions. The experimental period was 77 days (from November 2016 to February 2017).

### 2.2. Fish and Experimental Design

Rainbow trout from a fish farm (Zarzalejo, Albacete, Spain) were transported alive to the Aquaculture Laboratory of Polytechnic University of Valencia and randomly distributed in experimental tanks. Prior to the feeding trial, all fish were acclimatized for two weeks and fed a standard diet provided by the fish farm. After these two weeks, the fish were weighed (13.4 ± 0.3 g) and distributed into groups of 60 animals per tank. All fish were weighed every 28 days approximately. Previously, fish were anaesthetized with 30 mg L^−1^ of clove oil (Guinama^®^, Valencia, Spain) containing 87% eugenol. The fish were not fed for one day before weighing.

In order to evaluate gene expression throughout the intestine tract at 11 weeks after the start of the experiment, three fish digestive tract samples per tank were taken. Euthanasia was performed by anesthetic overdose (benzocaine 60 ppm for 10 min), and then fish were dissected to obtain the digestive tract. Two different sections were collected, based on the separation in sections proposed by Venou et al. [44]: anterior tract (the upper intestine, section between the stomach’s pyloric sphincter and the first bend of the digestive tract) and posterior tract of the intestine (section after this narrowing and the anus, obtaining two replications (portions of 16 mm^2^) of each section per fish. The fragments of the sections were stored in RNA later^®^ (Qiagen, Valencia, Spain) for 24 h at 4 °C and subsequently at −20 °C until gene expression analysis.

### 2.3. Diets and Feeding

The formulation and proximate composition of experimental diets are shown in Table 1. The PP blend (WG and SBM) was included in experimental diets at three dietary levels such that 80% (FM20), 90% (FM10) and 100% (FM0) of the FM was replaced. FM20 was considered the control diet. The three experimental diets were formulated to contain 44% crude protein (CP) and 19% crude lipid (CL), similar to commercial trout diets that contain CP levels ranging from 42% to 48% depending on fish size [45] and 19% CL [36]. Each diet was assayed in triplicate tanks and randomly assigned to 9 tanks. Diets were supplemented with feed-grade Lys, Met, valine (Val) and Tau based on the nutritional requirements for trout recommended by NRC [46]. The profile of dietary amino acids is shown in Supplementary Table S1.

Diets were prepared by cooking extrusion processing with a semi-industrial twin-screw extruder (CLEXTRAL BC-45, Firminy, St Etienne, France). The processing conditions were as follows: 100 rpm speed screw, temperature of 110 °C, 40–50 atm pressure and pellets with diameters from 2 to 3 mm. Experimental diets were assayed in triplicate. Fish were fed by hand twice a day (9:00 and 17:00 h) from Monday to Saturday until apparent satiation. Pellets were distributed slowly, allowing all fish to eat, and the total amount of feed distributed was recorded.

### 2.4. Analysis of Intestinal Health Status

In order to evaluate the effect of high levels of FM substitution on rainbow trout intestinal health, intestinal histology, the gene expression of inflammatory and immune processes and epithelial integrity in the anterior and posterior intestine was analyzed.

#### 2.4.1. Intestinal Histology

At the end of the experiment, intestine samples from five fish from each tank were collected and dissected into small pieces and preserved in phosphate-buffered formalin (4%, pH 7.4). All of the formalin-fixed tissues were routinely dehydrated in ethanol, equilibrated in ultraclean and embedded in paraffin according to standard histological techniques. Transverse sections were cut with a Microtome Shandon Hypercut to a thickness of 5 μm and stained with Alcian blue for gut examination. A total of eighteen sections per treatment were examined under a light microscope (Eclipse E400 Nikon, Izasa S.A., Barcelona, Spain).

For the measurements and observations of the intestine, a combination of criteria reported by several authors was followed [9,20], and the following parameters were measured: serous layer (SL), muscular layer (ML), submucous layer (SML), villi length (VL), villi thickness (VT), intra villi space and lamina propria length (LP) and thickness. In addition, a quantification of goblet cells (GC) was performed by counting the number of GC present in each villus. A total of six villi per section were used.

#### 2.4.2. Gene Expression

##### RNA Extraction

Total ribonucleic acid (RNA) was extracted from the anterior and posterior section of the intestine by traditional phenol/chloroform extraction, using the Trizol reagent (Invitrogen, Barcelona, Spain), and then purified and treated with DNase I using the NucleoSpin^®^ RNA Clean-up XS kit (Macherey-Nagel, Düren, Germany), according to the manufacturer’s instructions. The concentration, quality and integrity of the total RNA were evaluated with a NanoDrop 2000C spectrophotometer (Fisher Scientific SL, Madrid, Spain). Only samples that obtained an absorbance ratio A260/280 between 1.8–2.0 and A260/230 greater than 2.0 were included in the analysis. The RNA samples were stored at –80 °C until the stage of complementary deoxyribonucleic acid synthesis (cDNA) to avoid RNA degradation.

Subsequently, the cDNA was synthesized from 1 μg of RNA using the qScript Flex cDNA kit (Quanta BioScience, Beverly, MA, USA), according to the manufacturer’s instructions and using the Applied Biosystems 2720 thermal cycler. The thermocycler conditions were 22 °C during 5 min, 42 °C for 30 min and 85 °C for 5 min. Once the cDNA was obtained, it was stored at –20 °C until the gene expression was analyzed.

#### 2.4.3. Quantitative Real-Time RT-PCR (qPCR)

The real-time polymerase chain reaction (RT-qPCR) consists of the amplification of cDNA prepared using the reverse transcription (RT) of messenger ribonucleic acid (mRNA) by way of quantitative real-time polymerase chain reaction (qPCR), which is a tool commonly used to study and evaluate gene expression [48]. 

##### Reference and Target Genes

In order to select the best reference gene for the experiment, two candidate reference genes were selected: *Elongation Factor 1α* (*ELF-1α*) and *β-actin*, whose primer sequences are shown in Table 2. The stability was analyzed thanks to the BestKeeper program [49] based on the Ct values obtained. The expression of three inflammatory genes related to primary inflammation, *Interleukin 1β* (*IL-1β*)*, Interleukin 8* (*IL-8*) and *Transforming Growth Factor beta* (*TGF-β*), was analyzed. These inflammation-stimulating molecules are immuno-regulatory genes commonly used in rainbow trout. *IL-1β* was the first cytokine cloned in fish and has been identified in several teleost species, including salmonids [50]. It has a wide range of target cells and plays a fundamental and central role both in the initiation and in the regulation of inflammation [51]. *IL-8* is another pro-inflammatory cytokine that is involved in chemotaxis and in the activation of the different cell types involved in inflammation. It is known especially for being able to promote the adhesion of the monocytes and neutrophils that are in the blood circulation to the endothelial cells that form the blood vessels, helping them to pass from the blood to the inflamed tissue, so that they can exercise their action [52]. *TGF-β* is also another cytokine involved in immune and inflammatory processes, since it regulates the proliferation of the cells of the defense system (T and B lymphocytes), as well as the expression of some immunoglobulins. In addition, it regulates the expression of adhesion molecules. This molecule also acts as a chemoattractant for fibroblasts, monocytes and neutrophils [53]. It is believed that these three types of cytokines contribute to the hosting of defense mechanisms in response to colonization or bacterial invasion [54].

We also studied three immune genes related to the later response of the immune system: *Transferrin*, *Immunoglobulin T (IgT)* and *Interferon gamma (IFN-γ). Transferrin* is a lectin that binds to iron and has antimicrobial properties and, therefore, plays an important role in the pathology of many bacterial infections, limiting the amount of this essential endogenous nutrient available to invading pathogens and therefore, its ability to reproduce [55]. *IgT*, on the other hand, is an immunoglobulin specialized in immune responses of the intestinal mucosa [56]. Finally, *IFN-γ* is considered an important pro-inflammatory cytokine that plays an important regulatory role in both the innate and adaptive immune response in teleost cells [57], since it plays an important role in the activation of macrophages by increasing their phagocytic capacity. The primers used for the amplification reaction are shown in Table 2.

##### RT-qPCR Conditions and Gene Expression Quantification

All qPCR assays and expression analyses were performed using Real-Time PCR Applied Biosystems 7500 with SYBR^®^ Green PCR Master Mix (Thermo Fisher Scientific, Waltham, MA, USA) as a detection system. After initial activation of the Taq polymerase at 95 °C for 10 min, 42 cycles of PCR were performed using the Light Cycler with the following cycle conditions: 95 °C for 10 s and 60 °C for 30 s. To evaluate the specificity of the assay, a melting curve analysis was performed directly after the PCR by slowly increasing the temperature (1 °C min^−1^) from 60 to 95 °C, with a continuous record of the changes in the intensity of the fluorescent emission.

These assays were carried out in 96-well plates, and each reaction was carried out in duplicate. The volume per well was 20 μL, of which 5 μL were cDNA (diluted 1:40), 10 μL SYBR Green PCR Master Mix, 1 μL per primer (forward and reverse, 5 mM), 1 μL of water and 2 μL of reference fluorophore ROX™ (6-carboxy-X-rhodamine, 100 nM). In addition, a calibrator and a blank were included in all the plates. The interplate coefficient of variation was 1.94%. The reference genes and those of interest in all the samples were analyzed in duplicate.

For the relative quantification of gene expression, the 2^−ΔΔCt^ method developed by Weltzien et al. [62] was used applying the Equation (1). The quantification of the expression of the gene of interest was expressed in relation to the expression of the selected reference gene. In addition, with the use of the calibrator that was included in all the plates, it was possible to minimize the differences in reaction performance that may exist between plates.

*E* = [2*Ct* (*C*) − *Ct* (*M*)] × [2*CtHK* (*M*) − *CtHK* (*C*)] (1)

where *E* is relative gene expression, *C* is the calibrator, *M* is the sample (gene of interest) and *HK* is the reference gene (*ELF*-*1α* and *β*-*actin*).

### 2.5. Statistical Analysis

Growth data, feed utilization and data obtained from histological parameters measurements were evaluated using a one-way analysis of variance (ANOVA), with initial live weight as covariate (Snedecor and Cochran, [63]). The Newman–Keuls test was used to evaluate specific differences between diets at a level of *p* = 0.05 (Statgraphics, Statistical Graphics System, Version Plus 5.1, Herndon, VA, USA).

To verify if there are significant differences in the gene expression between experimental groups, the Newman–Keuls test, a multivariate analysis of variance, was carried out, considering diet and different sections as factors: and the combination of both factors for each gene individually. The calculation was performed with a confidence interval of 95% (*p* ≤ 0.05), and the data are shown as the mean ± SEM (standard error of the mean). Statistical data analyses were performed using the software Statgraphics© Centurion XVI (Statistical Graphics Corp., Rockville, MO, USA).

## 3. Results

### 3.1. Growth, Survival and Food Intake

Survival at the end of the experiment was 93.3, 81.7 and 51.1% in fish fed FM20, FM10 and FM0 diets, respectively. The mortality in all experimental groups was not progressive but was caused by a punctual increase in nitrite levels at the end of the experiment (day 70 of the trial). As all experimental groups were reared under the same recirculation freshwater system, all fish were submitted equally to environmental conditions.

In addition, there were significant differences in growth, with a lower final weight and SGR for the fish fed with the FM0 diet (51.1 g and 1.73% day^−1^). The feed intake (FI) and the feed conversion ratio (FCR) also varied according to the treatment, being higher for the FM0 diet (2.1 g *per* 100 g fish^−1^ day^−1^) (Table 3), and consequently registering the lowest protein efficiency ratio (PER).

### 3.2. Intestinal Health Status

#### 3.2.1. Intestinal Histology

The intestine measurement parameters are shown in Table 4. FM0 registered lower VT, LP and higher GC numbers at proximal intestine (PI) parameters, confirming this higher number of GC in the distal intestine (DI). No significant parameters were observed in the rest of the proximal or distal parameters.

#### 3.2.2. Gene Expression

Both reference genes provided standard deviations lower than 1.00 [64]; therefore, we decided to use the geometric average of both genes as a normalization factor in the current study. The mean of both genes was 18.56 ± 1.42 (*β-actin*, Ct) and 18.15 ± 1.65 (*ELF-1α*, Ct). The results are shown in Supplementary Table S2.

The results of gene expression, after a multifactorial analysis, showed differences between the inflammatory genes *IL-8* and *IL-1β* and the immune system *IFN-γ* depending on the diet administered to the fish, but not depending on the region of the intestine in which said genes are expressed (Table 5). Likewise, if the combined effect is considered, significant differences can be observed in most of the genes at anterior section. 

**Figure 1:**
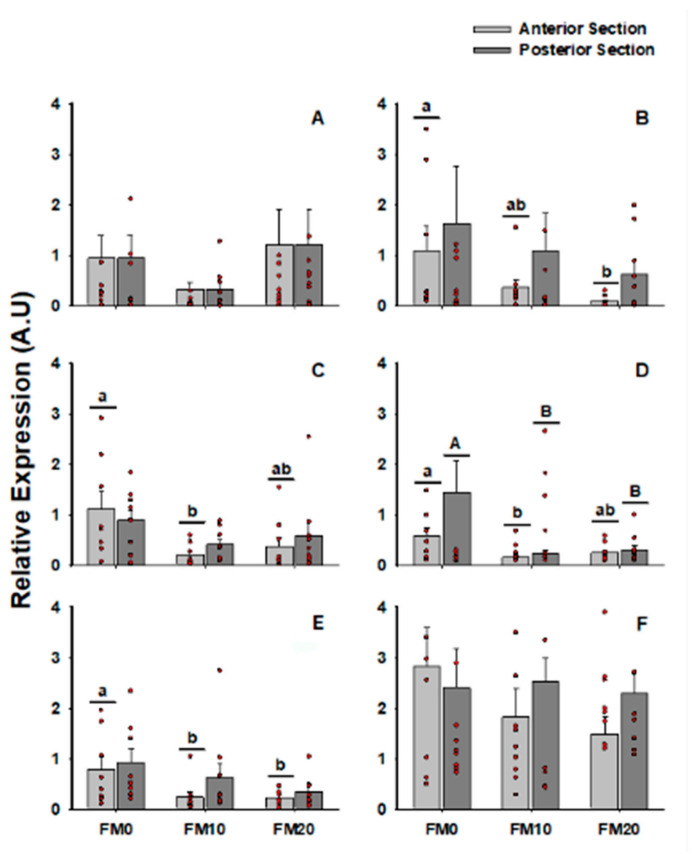
Gene expression of (**A**) *IgT*; (**B**) *Transferrin*; (**C**) *IFN-γ*; (**D**) *IL-8*; (**E**) *IL-1β* and (**F**) *TGF-β* genes taking into consideration intestinal section (anterior and posterior) and diet (FM0, FM10 and FM20). Lowercase letters reflect significant differences (*p* < 0.05) between diets in anterior section and capital letters reflect significant differences in posterior section.

The results of gene expression for *IgT, Transferrin, IFN-γ, IL-8, IL-1β* and *TGF-β* in different intestinal sections and experimental diets are shown in Figure 1. The FM0 diet induced, in general, higher expression, especially in anterior section. Fish belonging to the same diet group did not register differences between sections.

Considering only the intestinal section, no significant differences were observed (Supplementary Figure S1). However, the registered average was usually higher in the posterior section with respect to the anterior section for all the genes studied. As no relevant results were observed between intestinal sections, from this point on the analysis was performed without considering this factor.

Regarding diet, regardless of the intestinal section (Supplementary Figure S2), the fish fed the FM0 diet presented higher values than those fed the other diets. Significant differences were found in the expression of the *IFN-γ, IL-8* and *IL-1β* genes. Finally, independently of the diet, *TGF-β* showed higher levels than the rest of the target genes (Supplementary Figure S3). No differences were found in the rest of the target genes.

## 4. Discussion

Although a total FM replacement was not possible without a diminishment in growth and fish health, a higher FM substitution (90%) than in previous trials [36,37] was achieved, maintaining good intestinal health and productive performance.

The SBM and WG combination was adequate to meet fish nutritional requirements without affecting fish growth, possibly due to the fact that the diet contained sufficient AA for fish growth. Despite the feed supplementation with synthetic AA to cover trout needs, especially in the plant-based diet, often the availability of these differs depending on diet composition, such as the amount of fiber and ANFs. Lower AA availabilities may lead to growth deficiencies or health status, as occurs with a lack of methionine, lysine and/or cysteine [65], due to their relevance in protein synthesis in relation to immune system, such as cytokines [66]. Therefore, this possible lower availability could have induced the higher intake in the FM0 diet. In previous studies the high inclusion of vegetable sources decreased the food intake, mainly due to the diet palatability; however, in the present work it seems that WG and SBM did not affect the palatability, and the higher intake could be attributed to the need to satisfy their requirements in essential AA. Therefore, the FM0 diet possibly presented an inadequate AA profile, which was confirmed by the PER results, which were significantly lower in the FM0 experimental group, which showed the worst protein quality and therefore an unbalanced AA profile.

As in previous studies [2,5,9,34,35], a total FM replacement was not feasible due to the lower growth performance results and the induction of immune and inflammatory responses at intestinal levels. Other studies on trout achieved good results with much lower FM substitution levels, using PP such as fermented SBM (40%) [67], sunflower meal or SBM (40%) [68], enzyme-treated SBM powder (50%) [69] and a mixture of CG, WG, extruded peas and rapeseed meal (50%) [70]. Higher substitutions (around 20%) have been successfully achieved in this species [9,10,37], and FM inclusion is currently accepted for rainbow trout, even in commercial feeds, with 16% of FM inclusion [71]. Generally, levels of plant meals in salmon and trout feed formulations are limited by their composition (relatively low CP and high crude fiber content) and by the presence of ANFs and non-soluble carbohydrates [46,72]. Nowadays, trout feed has lower carbohydrate levels, higher digestibility and higher energy levels (e.g., 34–55% CP, 18–32% fat, 10–20% starch) [73]. Commercial diet formulations have achieved low FM content, up to 16% [71], using a combination of alternative protein sources and synthetic AA to better balance the AA levels [74]. An example is Tau, a synthetic AA included in the study. Tau is a determinant AA in some carnivorous species [75]. Rainbow trout have the capacity to synthesize Tau from cysteine (Cys) [76]; however, the rate of synthesis may be inadequate to fulfill the Tau needs of rainbow trout fed an all-plant protein diet [77]. Gaylord et al. [78] conducted a trial with plant diets supplemented with Tau, observing that Tau supplementation in an all-plant protein diet appeared critical for maintaining growth rates and FCRs equivalent to fish fed fishmeal-containing diets. In fact, supplementation with a 5 g kg^−1^ diet of Tau in a plant-based diet was sufficient to increase growth to levels equivalent to those seen in fish fed FM diets.

In our study, the results obtained clearly indicate that up to 90% of FM (FM10) can be substituted without presenting high mortality, an inflammatory process, or compromising the growth performance. The high mortality was registered after an accidental increase in the level of nitrite at the end of the experiment. This increase in nitrites was consistent across all the tanks; therefore, it affected all experimental groups equally, so differences in eventuality may be attributed to an imbalance in diet, intestinal inflammation or an altered immune system.

It has been shown that PP sources can induce intestinal inflammation because many of them contain various ANFs, especially legumes [72]. The negative effect of SBM on growth has been attributed to ANFs such as protease inhibitors, tannins, lectins and non-starch polysaccharides [79]. In the current study, the higher VT and LP values at PI were registered for the FM0 experimental group, probably due to the high content of these ANFs. The opposite trend seems to be registered at DI, lower VT and LP values, but without significant differences.

Similar inflammatory responses against FM substitution with plant diet has also been reported in salmonids [80], due to the infiltration of inflammatory cells, such as lymphocytes, macrophages, eosinophils and neutrophil granular cells, and diffuse immunoglobulin M (IgM). At FM replacement levels greater than 50%, rainbow trout and Atlantic salmon fed SBM-containing diets exhibited a progressive decline in growth rate, which was accompanied by a corresponding depression of non-specific immune capacity and exacerbation of pathological changes in the DI [11,18,81,82]. SBM levels in diets of 400–450 g kg^−1^ seem to be the inclusion limit, beyond which intestinal damage occurs in trout [3,40]; this can explain the VT of proximal intestine differences and the relatively higher surface in the fish fed the FM0 diet, for which SBM was included at 400 g kg^−1^.

Furthermore, in the present study a lower GC number was found in both PI and DI of fish fed FM0 diet. A reduction in GC might denote an immunosuppression in rainbow trout fed diets without FM, such has been shown in previous studies carried out on rainbow trout fed PP for 63 days [83]. Goblet cells maintain the epithelial homeostasis through the secretion of a mucosal barrier that acts as a lubricant, preserving the epithelium, although some studies support that GC can act as a major cellular component of the innate and adaptative defense system [84]. In addition, a lower number of GC was also observed in other species such as gilthead seabream when fed with diets comprising 100% PP sources in substitution of FM or high PP levels [85]. The dietary effect on goblet cells may be caused by phytate or fibers contained in protein sources rather than other ANFs that can be eliminated by heat treatment [86].

In other studies, it has been proven that the use of SBM as a protein source also induces immune alterations. In Atlantic salmon (*Salmo salar*) the inclusion of 10% SBM produces detrimental inflammatory effects at the intestinal level [84], and a high dietary inclusion level of PPC (35%) resulted in significant adverse effects on growth performance, nutrient digestibility, digestive physiology and gut health [43]. High concentrations of dietary soybean suppress salmonid growth rates and non-specific immune capacity. Burrells et al. [18] found that the immunosuppression became evident at protein soybean products inclusion rates of 60–70% in rainbow trout, which was coincident with a reduction in weight gains and the appearance of demonstrable pathological changes in the DI. Similarly, Jalili et al. [42] formulated diets for rainbow trout with different levels of FM substitution with vegetable protein sources (WG, CG and SBM) and according to their findings, higher PP inclusions (70 and 100%) resulted in undesirable effects on growth, nutritional indices, serum total immunoglobulin and alternative complement activity. Finally, they concluded that FM levels lower than 20% were not able to maintain growth rates and/or growth efficiencies with good health status [34,35,38].

Reinforcing the theory of altered intestinal health caused by a total FM substitution, higher expression of immune and inflammatory genes was reported in FM0 group, with high expressions of the genes *IL-1β, IL-8* and *IFN-γ*. This is consistent with the results observed in previous studies [25,87]. In particular, *IL-1β* and *IL-8* are two complementary pro-inflammatory cytokines, generally induced in the early stage of an immune response [88]. While *IL-1β* induces the growth and proliferation of T and B lymphocytes and macrophages [89], *IL-8* has chemotactic capacity and attracts neutrophils to the site of possible infection [90]. The production of *IL-8* is stimulated by the expression of *IL-1β*, so it is not surprising to see the simultaneous expression of these two cytokines and that similar patterns are exhibited. Regarding *IFN-γ*, it is also a cytokine of the immune system, whose role is the activation of macrophages, thus increasing their phagocytic capacity [57]. The increase in its gene expression in relation to the pro-inflammatory mediators *IL-1β* and *IL-8* has been reported as a mechanism of regulation of inflammation [87] and the activation of innate immunity in response to a possible infection [91]. Interestingly, they share the same expression pattern, which may also be because they are the same type of protein.

No significant differences were observed between the two sections for any of the genes studied. Unlike the present results, there are many other studies in which the posterior intestine is recognized as the site with the greatest inflammatory impact [3], and it is precisely the zone where there is a greater number of associated immune cells [92], and where the local immune response is usually higher [93]. In addition, the higher expression levels of the *TGF-β* gene in both sections, compared with the rest of the genes, could be explained by the fact that this molecule fulfills a function both inhibiting and stimulating inflammation and, in addition to these antagonistic functions, it also exerts profound effects on immune cells, including lymphocytes and macrophages [94].

Considering the interaction between both factors, higher values were again recorded for the FM0 group, mainly in the anterior section, with the exception of *IgT* and *TGF-β*. This work shows opposite results to those observed in previous studies [87,95], in which the inclusion of vegetable proteins in the diet produced an increase in the expression of this specialized molecule in immune responses of the intestinal mucosa [56]. Regarding *Transferrin,* which is a plasma protein involved in iron supply, it creates an environment in the mucosa that is not suitable for bacterial survival when it binds to it. It functions as a response mechanism to the management of environmental stress and plays an important role in the innate immune system [96]. So, the higher level of PP inclusion in the FM0 group could be determining an intestinal inflammatory reaction that for a prolonged period may be responsible for the appearance of a certain local immunosuppression, causing an immune dysfunction, and, therefore, fish more susceptible to stress situations [25]. Thus, the alteration of inflammatory and immune intestinal status may have contributed to the lower survival of the FM0 group after the nitrite peak event.

Therefore, possible reasons for the lower growth and survival may be due to different factors or their combination. Besides the possible deficiency of some AA, the higher mortality observed in the FM0 group may be caused by the combination of histological intestinal alteration possibly induced by ANFs present in vegetable raw material, causing inflammation and maybe a certain level of immunosuppression [18,81].

## 5. Conclusions

The results of this study show that 90% of FM substitution is feasible using a PP blend of WM, WG and SBM supplemented with Tau, Val, Lys and Met without apparent decrement in growth or intestinal health status.

## Figures and Tables

**Table 1:** Formulation and proximate composition of experimental diets.

	Experimental Diets		
	FM0	FM10	FM20
**Ingredients (g kg^−1^)**			
Fishmeal ^1^	0	100	200
Wheat meal ^2^	116	158	201
Wheat gluten ^3^	214	201	180
Soybean meal ^4^	400	300	200
Soybean oil	88	89	90
Fish oil	100	91	82
Calcium phosphate	38	33	28
Taurine ^5^	20	10	5
Valine ^5^	2	0	0
L- Methionine ^5^	4	2	0
L-Lysine Clh ^5^	8	6	4
Vitamin-mineral mix ^6^	10	10	10
**Proximate composition (g kg^−1^ on dry matter)**			
Dry matter (DM)	913	912	912
Crude protein (CP)	446	440	434
Crude lipid (CL)	191	190	192
Ash	42	68	75
**Calculated values**			
Carbohydrates (CHO) ^7^	322	302	299
Calculated GE (MJ kg^−1^) ^8^	22.5	22.6	23.3

^1^ Fishmeal: (93.2% DM, 70.7% CP, 8.9% CL, 15.1% Ash); Vicens I Batllori S.L., Girona, Spain. ^2^ Wheat meal (92.4% DM, 17.1% CP, 2.4% CL, 78.3% CHO, 2.4% Ash); Piensos Y Cereales Desco, Museros, Valencia, Spain. ^3^ Wheat gluten: (70.9% CP, 1.3% CL, 34.1% CHO, 1.5% Ash); Dadelos Agrícola, Valencia, Spain. ^4^ Soybean meal: (88.2% DM, 49.9% CP, 2.2% CL, 7.1% Ash); Piensos Y Cereales Desco, Valencia, Spain. ^5^ Taurine, Valine, L-Methionine and L-Lysine: Guinama S.L.U. ^6^ Vitamin and mineral mix (values are g kg^−1^ except those in parentheses): Premix: 25; Choline, 10; DL-α-tocopherol, 5; ascorbic acid, 5; (PO_4_)_2_Ca_3_, 5. Premix composition: retinol acetate, 1,000,000 IU kg^−1^; calcipherol, 500 IU kg^−1^; DL-α-tocopherol, 10; menadione sodium bisulphite, 0.8; thiamine hydrochloride, 2.3; riboflavin, 2.3; pyridoxine hydrochloride, 15; cyanocobalamine, 25; nicotinamide, 15; pantothenic acid, 6; folic acid, 0.65; biotin, 0.07; ascorbic acid, 75; inositol,15; betaine, 100; polypeptides 12. Zn, 5; Se, 0.02; I, 0,5; Fe, 0.2; CuO, 15; Mg, 5.75; Co, 0.02; Met, 1.2; Cys, 0.8; Lys, 1.3; Arg, 0.6; Phe, 0.4; Trcp, 0.7; excpt. 1000 g. ^7^ Carbohydrates (g kg^−1^) = 100−CP (g kg^−1^)−CL (g kg^−1^)−Ash (g kg^−1^). ^8^ Calculated energy (MJ kg^−1^) = [(51.8 × C)–(19.4 × N)]. The C–N was analyzed by way of the Dumas principle (TruSpec CN; Leco Corporation, St. Joseph, MI, USA). Calculated according to Brouwer [47].

**Table 2:** General information about the target and reference genes.

Category	Gen/Protein Description	Abbrev.	Primer Sequence		GenBank	BP Size	Reference
			Forward	Reverse			
Housekeeping genes	*Beta-actin*	*β-actin*	GCCGGCCGCGACCTCACAGACTAC	CGGCCGTGGTGGTGAAGCTGTAAC	EZ908974	73	Evenhuis and Cleveland, [58]
	*Elongation factor 1-alpha*	*ELF-1α*	ACCCTCCTCTTGGTCGTTTC	TGATGACACCAACAGCAACA	AF498320	63	Kania et al. [59]
	*Transferrin*		5’CCACCTCCAGGGCCATTAAATG3’	5’ATCCACCGCTATGGCATCTGCC3’	D89083		Talbot et al. [60]
Immune	*Immunoglobulin T*	*IgT*	AACATCACCTGGCACATCAA	TTCAGGTTGCCCTTTGATTC	AY870265	80	Evenhuis and Cleveland, [58]
	*Interferon gamma*	*IFN-γ*	CTGTTCAACGGAAACCCTGT	AACACCCTCCGATCACTGTC	NM 001160503	62	Evenhuis and Cleveland, [58]
	*Interleukin-1 beta*	*IL-1β*	ACATTGCCAACCTCATCATCG	TTGAGCAGGTCCTTGTCCTTG	AJ223954	91	Pérez-Sánchez et al. [61]
Inflammatory	*Interleukin 8*	*IL-8*	CTCGCAACTGGACTGACAAA	TGGCTGACATTCTGATGCTC	AJ279069	148	Evenhuis and Cleveland, [58]
	*Transforming growth factor beta*	*TGF-β*	TCCGCTTCAAAATATCAGGG	TGATGGCATTTTCATGGCTA	X99303	71	Evenhuis and Cleveland, [58]

**Table 3:** Effect of the different diets on growth and nutritive parameters in rainbow trout.

	Experimental Diets			
	FM0	FM10	FM20	*p*-Value
Initial weight (g)	13.60 ± 0.23	13.43 ± 0.23	13.13 ± 0.23	0.3911
Final weight (g)	49.07 **^b^** ± 1.69	72.94 **^a^** ± 1.69	72.24 **^a^** ± 1.69	0.0001
Survival (%)	51.11 **^b^** ± 5.62	81.67 **^a^** ± 5.62	93.33 **^a^** ± 5.62	0.0046
SGR ^1^ (% day^−1^).	1.73 **^b^** ± 0.04	2.24 **^a^** ± 0.04	2.21 **^a^** ± 0.04	0.0043
FI (g 100 g fish^−^^1^ day^−^^1^) ^2^	2.10 **^a^** ± 0.06	1.85 **^b^** ± 0.06	1.79 **^b^** ± 0.06	0.0188
FCR ^3^	1.52 **^b^** ± 0.06	1.09 **^a^** ± 0.06	1.01 **^a^** ± 0.06	0.0018
PER ^4^	1.62 **^b^** ± 0.09	2.31 **^a^** ± 0.09	2.49 **^a^** ± 0.09	0.0025

^1^ SGR, Specific growth rate = 100 × ln (final weight/initial weight)/days. ^2^ FI, feed intake = 100 × feed consumption (g)/average biomass (g) × days. ^3^ FCR, feed conversion ratio = feed consumption (g)/biomass gain (g). ^4^ PER, protein efficiency ratio = biomass gain (g)/protein intake (g). Means of triplicate groups. Values are presented as mean ± SEM (*n* = 3). Values in the same row with different superscript letters are significantly different (*p* < 0.05). Initial weight was considered as covariable for final weight and SGR.

**Table 4:** Effect of the different diets on proximal and distal measurements in rainbow trout.

	Experimental Diets			
	FM0	FM10	FM20	*p*-Value
**Proximal Intestine**				
VL (µm)	782.8 ± 77.6	831.6 ± 56.0	715.2 ± 47.5	0.2850
VT (µm)	209.9 **^b^** ± 14.1	164.6 **^a^** ± 10.2	149.8 **^a^** ± 8.6	0.0026
LP (µm)	53.7 **^b^** ± 4.5	35.4 **^a^** ± 3.3	33.7 **^a^** ± 2.8	0.0012
GC	2.7 **^a^** ± 1.5	8.9 **^b^** ± 1.0	15.2 **^c^** ± 0.9	0.0000
SL (µm)	67.8 ± 5.3	55.3 ± 4.7	62.7 ± 4.6	0.2184
ML (µm)	152.7 ± 13.0	117.3 ± 11.6	140.6 ± 11.4	0.1214
SML (µm)	60.8 ± 5.0	59.3 ±4.5	57.5 ± 4.4	0.8834
**Distal Intestine**				
VL (µm)	547.5 ± 134.3	754.2 ± 54.8	756.8 ± 60.0	0.3396
VT (µm)	135.91 ± 26.9	160.88 ± 11.1	168.4 ± 11.8	0.5404
LP (µm)	26.9 ± 3.7	31.0 ± 1.5	33.2 ± 1.6	0.2633
GC	2.1 **^a^** ± 2.8	9.4 **^b^** ± 1.1	14.0 **^b^** ± 1.2	0.0006
SL (µm)	70.9 ± 12.6	80.0 ± 7.0	67.3 ± 9.6	0.5428
ML (µm)	93.3 ± 15.8	110.1 ± 8.7	110.4 ± 11.9	0.6266
SML (µm)	66.2 ± 7.1	50.1 ± 3.9	56.3 ± 5.3	0.1437

VL, villi length; VT, villi thickness; LP, lamina propria; GC, goblet cells; SL, serous layer; ML, muscular layer; SML, submucous layer. Values are the mean ± SEM (*n* = 18). Different letters in the same line denote significant differences (*p* < 0.05).

**Table 5:** *p*-values obtained after multifactor analysis.

	*IgT*	*Transferrin*	*IFN-γ*	*IL-8*	*IL-1β*	*TGF-β*
Function	Immune system	Immune system	Immune system	Inflammatory	Inflammatory	Inflammatory
Section	0.1966	0.2457	0.7111	0.1358	0.201	0.4442
Diet	0.1905	0.2857	0.0042 *	0.0088 *	0.018 *	0.4428
Interaction (section × diet)						
FM0-Ant vs. FM10-Ant vs. FM20-Ant	0.2137	0.0608	0.0177 *	0.014 *	0.0271 *	0.2446
FM0-Pos vs. FM10-Pos vs. FM20-Pos	0.4101	0.6864	0.2198	0.0512	0.2386	0.9588
FM0-Ant vs. FM0-Pos	0.8031	0.8249	0.5844	0.2244	0.7244	0.6994
FM10-Ant vs. FM10-Pos	0.1086	0.3628	0.1074	0.1703	0.2292	0.3474
FM20-Ant vs. FM20-Pos	0.2350	0.0620	0.5153	0.6035	0.3455	0.1546

Values with asterisk indicate significant differences were found (*p* < 0.05).

## Data Availability

The data presented in this study are available on request from the corresponding author.

## Supplementary Materials

The following are available online at https://www.mdpi.com/article/10.3390/ani11123577/s1, Supplementary Figure S1: Gene expression of *IgT, Transferrin (Transf), IFN-γ, IL-8, IL-8, IL-1β* and *TGF-β* in anterior and posterior intestine section, Supplementary Figure S2: Gene expression of *IgT, Transferrin (Transf), IFN-γ, IL-8, IL-1β* and *TGF-β* in each experimental diet: FM0, FM10 and FM20. Different letters reflect significant differences (*p* < 0.05), Supplementary Figure S3: Gene expression of *IgT, Transferrin (Transf), IFN-γ, IL-8, IL-1β* and *TGF-β* based on experimental diet: FM0, FM10 and FM20. Different letters reflect significant differences (*p* < 0.05) in the expression, Supplementary Table S1: Amino acid profile of experimental diets expressed in g per 100 g of wet matter, Supplementary Table S2: Results from BestKeeper program analysis.
