# Supplementary material for: Successful Inclusion of High Vegetable Protein Sources in Feed for Rainbow Trout without Decrement in Intestinal Health
Source: Animals (Basel). 2021 Dec 16;11(12):3577. doi: 10.3390/ani11123577 (PMC8698200; doi:10.3390/ani11123577)
Supplement: Supplementary file 1 [file animals-11-03577-s001.zip › Supplementary Table S1.pdf]

Supplementary Table S1. Amino acid profile of experimental diets expressed in g per 100 g of wet matter.

Amino acids (%wet weight)	FM0	FM10	FM20
***EAA*[1] g / 100g**			
Histidine	1.32	0.87	1.04
Arginine	6.52	2.99	3.22
Valine	2.33	1.59	1.74
Methionine	0.70	0.67	0.54
Lysine	2.90	2.74	2.14
Isoleucine	1.88	1.35	1.47
Leucine	3.25	2.32	2.55
Phenylalanine	2.12	1.34	1.55
Threonine	1.79	1.22	1.41
***NEAA*[2]**			
Aspartic	4.12	3.08	3.13
Serine	2.77	1.79	1.96
Glutamic	13.94	9.77	9.53
Glycine	2.17	1.61	1.94
Alanine	1.75	1.42	1.60
Proline	4.60	3.36	3.53
Cysteine	0.65	0.73	0.53
Tyrosine	1.42	0.89	1.00
