# Supplementary material for: Successful Inclusion of High Vegetable Protein Sources in Feed for Rainbow Trout without Decrement in Intestinal Health
Source: Animals (Basel). 2021 Dec 16;11(12):3577. doi: 10.3390/ani11123577 (PMC8698200; doi:10.3390/ani11123577)
Supplement: Supplementary file 1 [file animals-11-03577-s001.zip › Supplementary Table S2.pdf]

Supplementary Table S2. Results from BestKeeper program analysis.

	β-actin	***ELF-1α***
N	92	92
geo Mean [Ct]	18.54	18.12
Mean [Ct]	18.56	18.15
min [Ct]	17.30	15.41
max [Ct]	20.99	20.03
std dev [± Ct]	0.51	0.72
CV [% Ct]	2.74	3.99
min [x-fold]	-2.37	-6.54
max [x-fold]	5.45	3.75
std dev [± x-fold]	1.42	1.65
coeff. of corr. [r]	0.885	0.942
