# Supplementary figures and images for: Successful Inclusion of High Vegetable Protein Sources in Feed for Rainbow Trout without Decrement in Intestinal Health
Source: Animals (Basel). 2021 Dec 16;11(12):3577. doi: 10.3390/ani11123577 (PMC8698200; doi:10.3390/ani11123577)

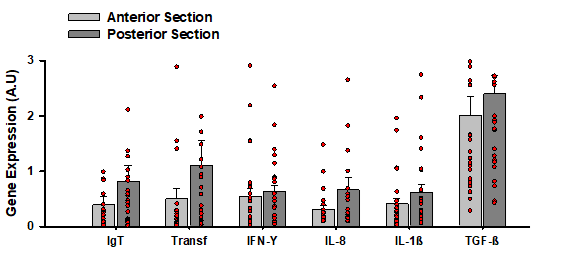

Supplement: Supplementary file 1 [file animals-11-03577-s001.zip › Supplementary Figure S1.png]

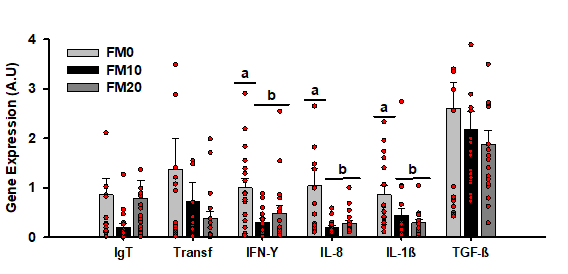

Supplement: Supplementary file 1 [file animals-11-03577-s001.zip › Supplementary Figure S2.png]

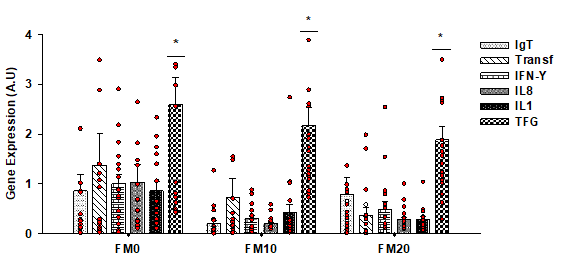

Supplement: Supplementary file 1 [file animals-11-03577-s001.zip › Supplementary Figure S3.png]
